# A hierarchical surrogate approach to biomass ethanolysis reaction kinetic modelling†

**DOI:** 10.1039/d4re00378k

**Published:** 2024-11-13

**Authors:** Ailís O'Shea, Conall McNamara, Prajwal Rao, Mícheál Howard, Mohammad Reza Ghanni, Stephen Dooley

**Affiliations:** a School of Physics, Trinity College Dublin Ireland aoshea2@tcd.ie; b Department of Chemical Sciences, University of Limerick Limerick Ireland; c School of Engineering, Trinity College Dublin Ireland

## Abstract

The reaction mechanism and kinetics of the sulfuric acid catalysed ethanolysis of glucose, cellulose, xylan, and corncob were investigated using a combination of experiments and empirical reaction mechanism modelling. The experimental study was carried out in ethanol at various temperatures between 150 °C and 200 °C. Ethanol mediates the depolymerisation and formation of ethyl levulinate from the carbohydrates in the substrates. Ethanol itself is converted to the corresponding ether in a parallel acid-catalysed condensation reaction. The complementary synergistic thermal and combustion properties of the main components in the resulting mixture, ethyl levulinate, diethyl ether, and ethanol, create the potential for the use of the product mixture as a tailored drop-in biofuel. The concentrations of the main species in the product mixtures from the reaction experiments were used to build a hierarchical surrogate kinetic model based on feedstock composition. The reaction mechanism provided to the surrogate kinetic model is informed by a comparative experimental mechanistic study of the ethanolysis of glucose and fructose. The study shows that the major reaction species formed from glucose ethanolysis are ethyl glucoside and ethyl levulinate, whereas fructose ethanolysis primarily forms 5-hydroxymethylfurfural, 5-ethoxymethylfurfural, ethyl fructoside and ethyl levulinate. The study shows that fructose produces a higher yield of ethyl levulinate than glucose and that fructose does so at a rate approximately ten times faster than glucose. The rate of formation of both ethyl levulinate and diethyl ether increases with increasing temperature. The maximum yields (mass%) of ethyl levulinate achieved from the ethanolysis of glucose, cellulose, xylan, and corncob are 39.3, 39.1, 7.9, and 18.6%, respectively. Ethyl levulinate yields reach a maximum steady state value for each feedstock that is independent of temperature. The conversion of the model compounds, glucose, cellulose, and xylan, to ethyl levulinate in the presence of ethanol and sulfuric acid is a catalytic process. However, for corncob, the yield of ethyl levulinate is dependent on the concentration of sulfuric acid in the reaction. This effect is also observed in the mass fraction of diethyl ether formed, indicating that the hydrogen cation supplied by sulfuric acid is not being fully replenished in the corncob ethanolysis process. A corncob : acid mass ratio of 10 : 1 is identified as a sufficient sulfuric acid concentration to achieve a maximum steady state yield of ethyl levulinate. An empirical analysis of the experimental data show that the apparent activation energies of the global reaction of glucose to ethyl levulinate and ethanol to diethyl ether are 21.5 and 23.0 kcal mol^−1^, respectively. The hierarchical surrogate kinetic model for the ethanolysis of corncob based on its composition of cellulose, hemicellulose, and lignin was developed and has an overall *R*^2^ value of 0.88. The model was exercised to predict the major trends of the reaction system at various hypothetical conditions, demonstrating its utility as tool for process development.

## Abstract 2

The acid-catalysed ethanolysis of glucose, cellulose, xylan, and corncob is demonstrated at varying temperatures. A surrogate kinetic model captures reaction trends, providing a framework for biomass ethanolysis.

## Graphical abstract

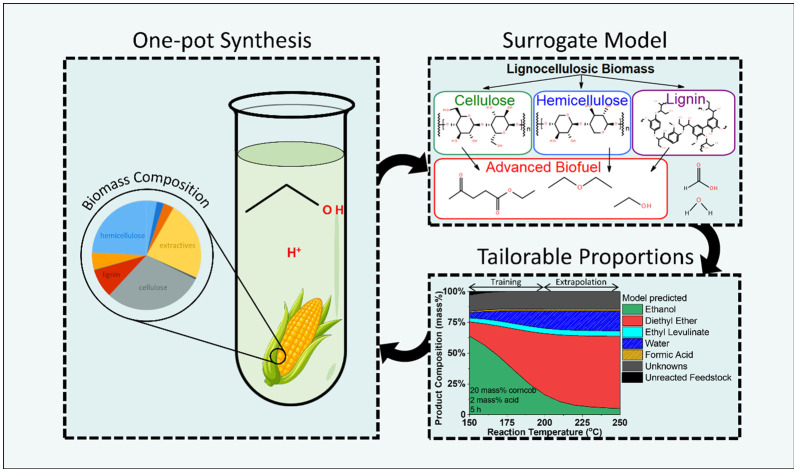

## 1. Introduction

In 2022 global CO_2_ emissions from the transport sector grew by more than 250 Mt CO_2_ to nearly 8 Gt CO_2_, 3% more than in 2021.^1^ The replacement of fossil derived fuels with biofuels will have an important role in the defossilisation of the global transportation industry. In the near term, commercialising low carbon footprint biofuels will be necessary for emission reductions in the existing ground transportation fleet and in the long term for the hard-to-abate haulage, shipping, and aviation sectors, where energy density is a defining factor.^2^

Conventional (1st generation) biofuels are derived from food crops grown on arable land. While conventional biofuels contribute to emission reductions, they face issues such as land-use change and competition with food resources. To circumvent such drawbacks, the development of advanced (2nd generation) biofuels derived from non-food/feed biomass, wastes and agricultural residues, as defined in Annex IX of the EU revised renewable energy directive (RED II), is a priority.^3^ RED II mandates member states to source ≥3.5% share of transportation energy from advanced biofuels and biogas. In the U.S., the renewable fuel standard is a federal program that requires transportation fuel sold in the United States to contain a minimum volume of renewable fuels, including specific targets for advanced biofuels. These volume requirements are established by the Environmental Protection Agency and are subject to adjustment based on various factors such as market conditions, technology advancements, and statutory requirements.

Advanced biofuels must compete with existing fossil fuels and conventional biofuels both technically and economically. While advanced biofuels face challenging technical circumstances due to their inferior elemental composition, and chemically recalcitrant nature of the feedstocks. However, lignocelluloses have the advantage of high sustainability, high availability, and low-cost. Configuring a viable route to advanced biofuels is a crucial for the net-zero transition and must be urgently addressed.

Several conversion processes for fuel production from lignocellulose are currently being explored including acid hydrolysis,^4^ alcoholysis,^5^ pyrolysis,^6^ gasification,^7^ hydrothermal liquefaction^8^ and hydrothermal carbonisation.^9^ Alkyl levulinates have been identified as potential drop-in diesel and gasoline biofuels,^10^ but also have applications such as green solvents, flavouring agents, lubricants, fragrances, and polymer plasticizers.^11,12^ Alkyl levulinate can be synthesised by alcoholysis in the presence of an acid catalyst. The acid alcoholysis reaction is analogous to that of the well-studied acid hydrolysis reaction where the alcohol solvent additionally functionalises levulinic acid analogues to produce alkyl levulinate. However, in addition to the formation of alkyl levulinate, the alcohol conversion to its corresponding dialkyl ether is usually not considered but is in fact a dominant process resulting in large amounts of ether. Although the largest component of the product mixture, the conversion of the alcohol to dialkyl ether is rarely investigated as part of the study of alcoholysis. Where dialkyl ether is studied, it is not investigated relative to alkyl levulinate production, other than by McNamara *et al.*^13^

Importantly, all the compounds present in the alcoholysis product, the alkyl levulinate, the alcohol and its dialkyl ether, are viable biofuel compounds with complementary synergistic properties.^14^ With knowledge of the reaction kinetics of the system and the combustion properties attributed to the product mixture by each of the molecular species in the evolving synthetic system, the rate and yield of the fuel synthesis can be simultaneously optimised, delivering drop-in mixtures of specified fuel and combustion properties. The direct alcoholysis of lignocellulose biomass can potentially minimise processing steps and thus offers a greater potential as a cost-effective process. To further investigate its viability, the reaction kinetics of the system as a whole must be interrogated.

To effectively produce alkyl levulinates from lignocellulosic biomass it is important to understand its chemical composition along with the variability in composition between feedstocks.

Lignocellulosic biomass is composed of cellulose, hemicellulose and lignin in varying amounts depending on the plant type, region of origin and growth season.^15^ It also contains moisture, extractives, and ash.^16,17^ The majority of alcoholysis studies to date have focused on the conversion of cellulose to platform chemicals. While cellulose is generally the largest component of biomass, hemicellulose and lignin make up a considerable portion of the overall mass. Hemicellulose is a heteropolysaccharide polymer composed of several different types of C_5_ and C_6_ sugar units such as xylose, arabinose, mannose, galactose and glucose. Xylan, typically the most abundant hemicellulose polymer in hardwoods and herbaceous biomasses,^18,19^ warrants investigation when considering the conversion of biomass feedstocks to platform chemicals but has not been studied as feedstock for one-pot alcoholysis to date and hence, xylan ethanolysis is a major focus of this paper. C_5_ sugars have been found to mainly form furfural, an intrinsic cause for the low yields of alkyl levulinates from biomass.^20^ C_5_ sugars can be converted to alkyl levulinates in a multi-step process. The initial acid-catalysed conversion to furfural, a partial hydrogenation of furfural to produce furfuryl alcohol, and the subsequent acid catalysed conversion of furfuryl alcohol into levulinic acid.^21–24^ In the one-pot alcoholysis process furfural is the main product with trace amounts of alkyl levulinate detected.^25^ Gomes *et al.* found the hemicellulose portion of sugarcane bagasse to have a positive influence on ethyl levulinate yield.^26^ Work by Xun Hu *et al.* utilised inclusion of a hydrogenation catalyst to greatly increase the yield of alkyl levulinate in the one-pot system with furfural, 2-(dimethoxymethyl)furan, and furfuryl alcohol as key intermediates.^27^ The hydrogenation step proceeds more efficiently in polar solvents (*i.e.*, ethanol, diethyl ether) than in non-polar solvents (*i.e.*, toluene), as the polar solvents tended to favour the hydrogenation of the furan ring in furfural over that of the carbonyl group in the same furfural.^28^ A more in-depth analysis of hemicellulose conversion to ethyl levulinate catalysed by sulfuric acid is required to model this system.

Consequently, the product composition from the ethanolysis process is dependent on the composition of the lignocellulosic biomass. Kinetic models describe the evolution of products in a system as a function of the reaction parameters in order to consider the optimal process configurations and technoeconomics. In the literature, the state-of-the-art kinetic models focus on ethanolysis of fructose,^29^ glucose^30–36^ and cellulose.^37^ There are no kinetic models on the ethanolysis of hemicellulose, lignin, or real-world biomass feedstocks. In addition to this, the study of the formation of diethyl ether is also neglected in the modelling literature. Literature models are also not mass conserved, usually only describing the mass that is measured.

In this work, a surrogate concept is set out to allow the kinetic model to predict product concentrations and kinetics for a wide range of feedstocks. Our surrogate concept uses the knowledge of model compound reactivity to hierarchically model real-world lignocellulosic biomass based on its biochemical composition. The mass conserved, hierarchical surrogate kinetic model, hence forth “surrogate kinetic model”, considers reaction is also integrated into the architecture as a universal sub-model. Importantly, in model training against measured species fractions, conservation of elemental mass is enforced, ensuring rigor to basic physics, and allowing prediction of species fractions that are not measured.

In the surrogate kinetic model, the rate of reaction is modelled according to an Arrhenius temperature dependence. Various sets of temperature dependent data afford an empirical method of estimating the activation energy of the observed reaction rates. The apparent activation energies of the global reactions to ethyl levulinate and diethyl ether can therefore be derived from temperature dependent kinetic data. An increase in temperature typically increases the rate of reaction. Additionally, the magnitude to which the rate of reaction is affected is dependent on the activation energy of a reaction. Thus, in a complex reaction system, the relative mass of products may be significantly influenced by the reaction temperature.

However, the steady state equilibrium mixture fraction results when a system reaches the species fraction configuration of lowest free energy, providing the maximum yield of ethyl levulinate possible at a given reaction condition. Knowledge of this quantity is important for the assessing the technoeconomic viability of the process in addition to anchoring any modelling to fundamental molecular thermodynamics. In the literature, yields are generally reported without information about whether the reaction is in steady state or kinetic phase. Thus, in this work the experimental reaction time is specifically pursued to the steady state regime of ethyl levulinate production.

In the work by McNamara *et al.*,^13^ it was deduced that ethyl levulinate production from biomass alcoholysis involves contributions from components other than cellulose, which challenges the common assumption in the literature. This study tests this deduction by utilising xylan as a model for hemicellulose, with the result informing if ethyl levulinate is also produced from the hemicellulose portion of biomass.

Additionally, while the work by McNamara *et al.* focused on a single temperature (150 °C) to determine optimal reaction conditions for ethyl levulinate production, this study investigates the effect of temperature on the production of ethyl levulinate, diethyl ether, ethanol and humins from the ethanolysis of glucose, cellulose, xylan, and corncob. By conducting experiments across a range of temperatures, the temperature dependence of the rate of formation of the various products and their steady state yields will be determined and subsequently learned by the surrogate kinetic model.

To inform the mechanistic construct of the surrogate kinetic model this work performs specific experiments to learn the comparative mechanism and kinetics of the ethanolysis of glucose (d-glucopyranose) and fructose (d-fructopyranose).

The ethanolysis of fructose is a well understood process both in water and in ethanol. In ethanol, the pathway to ethyl levulinate appears to proceed through 5-hydroxymethylfurfural and 5-ethoxymethylfurfural competing with an additional pathway through ethyl fructoside and 5-ethoxymethylfurfural.^29,38,39^ This process is analogous to that which occurs in water with the additional ethylation reactions. Furthermore, the proton H^+^ is solvated on ethanol rather than fructose, resulting in C_2_H_5_OH_2_^+^ as the catalytically active species. This increases the catalytic performance in ethanol compared to water.^40^

The ethanolysis of glucose is a well understood process in water, but not in ethanol. In aqueous systems, the glucose and fructose systems follow similar reaction pathways where glucose can be isomerised to fructose through a hydride transfer.^41,42^ However, fructose converts more readily than glucose to 5-hydroxymethylfurfural and levulinic acid, achieving significantly higher yields.^43–46^ Caratzoulas and Vlachos report that d-glucose dehydrates 40 times slower than fructose.^47^ Assary *et al.* calculated the free energy for protonating fructofuranose (8.0 kcal mol^−1^) to be nearly half that required for glucopyranose (15.0 kcal mol^−1^) in water.^48^ This initial protonation of the hexose sugar is proposed as the rate-limiting step in hexose dehydration to 5-hydroxymethylfurfural.^49^

Only a limited amount of experimental data is available comparing fructose to glucose ethanolysis under the same reaction conditions.^50–52^ Particularly, a key question in the literature is to what extent if any, is glucose transformation to fructose a significant process of glucose/cellulose ethanolysis. To allow more precise education on this, the ethanolysis reaction of fructose is studied at identical conditions.

All of this understanding is assembled into the first surrogate kinetic model self consistently and hierarchically describing the ethanolysis of glucose, cellulose, xylan, and corncob.

Finally, the model is demonstrated to assess the behaviour of the ethanolysis process as a function of temperature, feedstock loading, and acid loading, which are each important exemplar parameters for an industry ethanolysis process.

## 2. Experimental

### 2.1 Materials

All materials are purchased from commercial suppliers and used without further purification unless stated otherwise. Ethanol (≥99.8%), ethyl levulinate (99%), sulfuric acid (95–97%), anhydrous dimethyl sulfoxide (≥99.9%), anhydrous d-(−)-fructopyranose (99%), and diethyl ether (≥99.8%) are purchased from Sigma-Aldrich. Anhydrous d-(+)-glucopyranose (99%) and cellulose (microcrystalline) are purchased from Alfa Aesar. Ethyl-β-d-glucopyranoside (98%) is purchased from Carbosynth Ltd. Sodium hydrogen carbonate (≥99.7%) is purchased from Fisher Scientific. Metal autoclaves and 25 mL PTFE liners are purchased from Xiamen CRTOP Machine Co., Ltd.

Steamed corn on the cob (*Zea mays*) is purchased from a local supermarket. The cob is removed of kernels and any remaining non-lignocellulosic mass is washed. The corncobs are dried over several days in an oven at 70 °C until no further mass loss is observed. The dried corncobs are ground (Retsch RM100) into a powder that is sieved (Retsch mesh sieves) to a particle size of 100–125 μm.

### 2.2 Biochemical analysis of corncob

A biochemical analysis of dried ground corncob is performed by Celignis Analytical according to NREL procedure for determination of structural carbohydrates and lignin in biomass.^53^ The extractives are removed (Dionex ASE-200) and the water-soluble extractives are analysed for sugars. Acid hydrolysis is carried out and the hydrolysate is analysed on an ion chromatograph (Dionex ICS-3000) to determine the cellulose and hemicellulose content. The hydrolysate is analysed using UV-VIS spectrophotometry (HP 8452A) to determine the acid soluble lignin content. The hydrolysis residue is then incinerated to determine the Klason Lignin content. The ash content is measured after incineration (Nabertherm furnace).

### 2.3 Reactive experiments

(i) For the mechanistic study, glucose or fructose (0.1 g), ethanol (3.95 g), and sulfuric acid (0.02 g) with a total volume of 5 mL are added to glass pressure tubes with a magnetic stirrer. The pressure tubes are sealed using PTFE plugs and FETFE O-rings for pressure sealing up to 1.03 MPa and placed in an oil bath set to the reaction temperature. The mixtures are heated to a reaction temperature of 165 °C and allowed to react for 0.25 to 24 h. At the prescribed time, each tube is removed from the oil bath and immersed in a water bath to quench the reaction.

(ii) For the temperature dependent hierarchical study, metal autoclaves are required to safely operate at higher temperatures. Glucose, cellulose, xylan or corncob (1 g), ethanol (3.95 g), and sulfuric acid (0.05 g) with a total mass of 5 g are added to a 20 mL PTFE liner with a magnetic stirrer. The mixtures are heated to reaction temperatures of 150, 165, 180, 190 and 200 °C and allowed to react for 0.33 to 12 h. The PTFE liner is sealed in a 150 cm^3^ (external volume) metal autoclave and placed into an aluminium heating block preheated to the reaction temperature. The autoclave takes approximately 25 min to reach the reaction temperature. The temperature of the aluminium heat block is continuously regulated using a thermocouple connected to the heating plate. The heat block is a large heat sink, ensuring a stable and consistent temperature throughout the reaction. At the prescribed time, the metal autoclave is removed from the heating block and allowed to air cool to room temperature.

To investigate the extent of conversion during the non-isothermal heating period, an experiment with a reaction time of 20 minutes is carried out. This is performed at higher temperatures, where the initial heating phase constitutes a more significant portion of the overall reaction time. For instance, at 150 and 165 °C, the time to reach steady state is 8 hours and 24 hours respectively, making the non-isothermal heating period relatively insignificant in the overall conversion process. The temperature gradient of the heating period is incorporated into the surrogate kinetic model, discussed further in section 2.7.

When at room temperature, the reaction mixture is centrifuged at 5500 rpm for 5 minutes (Benchmark LC-8 centrifuge). The insoluble substance formed during the alcoholysis reactions is known as humins. Once separated, the supernatant is neutralised with sodium hydrogen carbonate (50 mg). The supernatant is prepared for gas chromatography on a gravimetric basis. An aliquot of the reaction mixture (250 mg) is diluted in dimethyl sulfoxide such that the mass ratio of dimethyl sulfoxide to reaction mixture is 400 : 1.

### 2.4 Ion chromatography

Ion chromatography analysis is performed for the mechanistic study only. A 25 mg portion of the sampled reaction media is diluted with 1.0 g of deionised water. An ion chromatography system (Dionex ICS3000) is utilised for the separation and quantification of fructose, glucose, 5-hydroxymethylfurfural and ethylated carbohydrates *via* a Dionex Carbopac PA1 column. In this analysis system, an anion exchange guard (4–50 mm) and analytical column (4–250 mm) are connected in series. This column is heated to 18 °C and the analytes are eluted using an isocratic flow of deionised water at a flow rate of 1.1 mL min^−1^ and detected using an electrochemical detector. The column is re-conditioned using a gradient flow of 0.4 mol L^−1^ NaOH and 0.24 mol L^−1^ CH_3_COONa for 3 minutes after each analysis.

### 2.5 Gas chromatography

Gas chromatography (Agilent 8860, GC-FID) is carried out on an Agilent DB-624 column with helium as the carrier gas (constant flow of 20 mL min^−1^) to measure ethanol, diethyl ether, and ethyl levulinate. The inlet temperature is maintained at 225 °C with the detector set to 300 °C. 1 μL of sample is injected at a split of 16 : 1. The oven conditions are: 45 °C for 4 minutes, 20 °C min^−1^ to 170 °C, 10 °C min^−1^ to 220 °C. The concentration of major products in the sample, including ethanol, diethyl ether, and ethyl levulinate is computed through calibration curves, see Fig. S1 in the ESI.†

Each experiment and analysis are performed in duplicate with the standard deviation shown as error bars in the results section. The uncertainties presented for all the steady state concentrations and yields are the standard deviations of all data points in the steady state. The yield is calculated as the mass of ethyl levulinate formed over the mass of feedstock added (glucose, cellulose, xylan or corncob):1
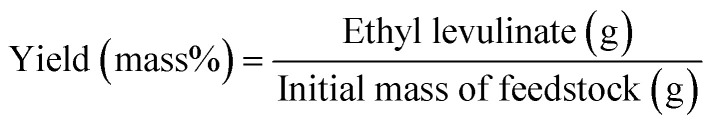


### 2.6 Activation energies

The temperature-dependant kinetic phase experimental data measured in this work is used to analytically determine the apparent activation energies of the assumed global reactions (eqn (2)–(5)) involved in the formation of ethyl levulinate and diethyl ether:2
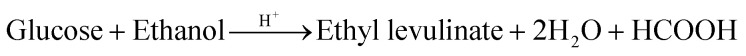
3
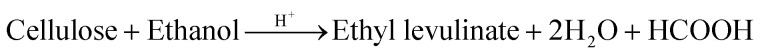
4
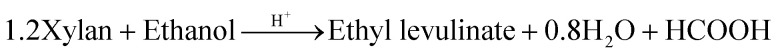
5
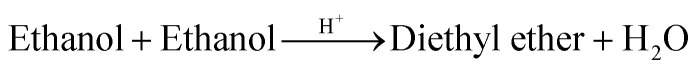
For each assumed global reaction, the measured increase in product formation per unit time from the kinetic phase experimental data allows for the determination of a value for the rate constant *k* at each temperature. By using the natural logarithm of the Arrhenius equation:6
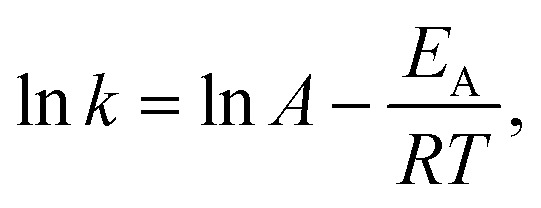
the apparent activation energies for reactions (2)–(5) are determined from the linear fit of ln *k vs. T*^−1^ (Fig. 6). These apparent activation energies are input into the surrogate kinetic model to integrate the thermodynamic information of model compounds, chemically representative to biomass molecular structures, into the model.

### 2.7 Surrogate kinetic model

#### Hierarchical reaction mechanism

**Fig. 1:**
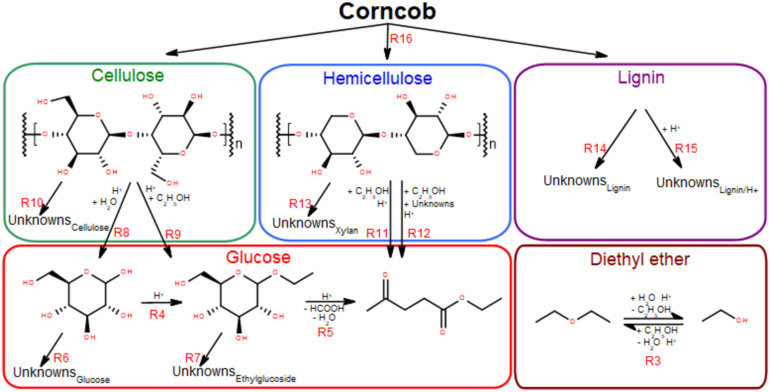
Reaction mechanism described by the surrogate kinetic model, depicting the reaction pathways and the hierarchical sub-models described in section 2.7. All reaction equations are listed in Table 1 (humins are the dark-coloured insoluble substance formed during the alcoholysis reactions. “Unknowns” are humins and other soluble species of unknown identity).

A surrogate kinetic model for the sulfuric acid catalysed ethanolysis of glucose, cellulose, xylan, and corncob is developed to provide a hierarchical molecularly consistent comprehension of the reaction mechanism and kinetics of the system. A reaction mechanism is proposed based on the analogous hydrolysis system and the major species detected in this study and in the pertinent computational and experimental literature.^54^ As the essential monosaccharide chemical structure is general to all biomasses, a concept of hierarchy is appropriate within the reaction mechanism – what occurs for glucose should occur for cellulose, should occur for lignocellulose. In this way, the information learned from the chemically discrete glucose can be transferred to the more complex but also chemically discrete cellulose, and those self-consistent learnings can be transferred to the less chemically discrete and much more complex lignocelluloses (corncob in this study). The surrogate reaction mechanism, shown in Table 1 and Fig. 1, is hierarchically and independently constructed and trained, one sub-model at a time: glucose, then cellulose, then xylan, then corncob. That is, the terms learned for the example of glucose, from glucose experiments are not subsequently adjusted in the training of the other sub-models.

**Table 1:** List of reactions in the surrogate kinetic model described in section 2.7 and their corresponding derived empirical kinetic parameters (in the ethanol sub-model, f and r denote the forward and reverse reactions for reversible reactions)

	#	Reaction	*A* (cm^3^ mol^−1^ s^−1^)	*E*_A_ (kcal mol^−1^)
Ethanol	1_f_	C_2_H_5_OH + H^+^ → C_2_H_5_OH_2_^+^	2.1 × 10^13^	5.7
	1_r_	C_2_H_5_OH_2_^+^ → C_2_H_5_OH + H^+^	2.1 × 10^10^	5.7
	2_f_	H_2_O + H^+^ → H_3_O^+^	1.0 × 10^12^	1.7
	2_r_	H_3_O^+^ → H_2_O + H^+^	1.0 × 10^12^	15.7
	3_f_	C_2_H_5_OH + C_2_H_5_OH_2_^+^ → C_2_H_5_OC_2_H_5_ + H_3_O^+^	1.3 × 10^16^	23.0^a^
	3_r_	C_2_H_5_OC_2_H_5_ + H_3_O^+^ → C_2_H_5_OH + C_2_H_5_OH_2_^+^	3.0 × 10^11^	23.0^a^
Glucose	4	C_6_H_12_O_6_ + C_2_H_5_OH_2_^+^ → C_8_H_16_O_6_ + H_3_O^+^	2.8 × 10^16^	21.5^a^
	5	C_8_H_16_O_6_ + C_2_H_5_OH_2_^+^ → C_7_H_12_O_3_ + HCOOH + H_2_O + C_2_H_5_OH_2_^+^	7.0 × 10^16^	21.5^a^
	6	C_6_H_12_O_6_ + H^+^ → Unknowns_Glucose_ + 3H_2_O + H^+^	5.0 × 10^14^	21.5
	7	C_8_H_16_O_6_ + H^+^ → Unknowns_Ethylglucoside_ + 4H_2_O + H^+^	4.1 × 10^13^	19.0
Cellulose	8	(C_6_H_10_O_5_)_*n*_ + H_3_O^+^ → C_6_H_12_O_6_ + H^+^	1.0 × 10^14^	23.8^a^
	9	(C_6_H_10_O_5_)_*n*_ + C_2_H_5_OH_2_^+^ → C_8_H_16_O_6_ + H^+^	2.7 × 10^17^	23.8^a^
	10	(C_6_H_10_O_5_)_*n*_ + H^+^ → Unknowns_Cellulose_ + 2H_2_O + H^+^	3.0 × 10^15^	23.2
Xylan	11	1.2(C_5_H_8_O_4_)_*n*_ + C_2_H_5_OH_2_^+^ → C_7_H_12_O_3_ + HCOOH + 0.8H_2_O	1.3 × 10^18^	24.6^a^
	12	1.2(C_5_H_8_O_4_)_*n*_ + Unknowns_Lignin/H^+^_ + C_2_H_5_OH_2_^+^ → C_7_H_12_O_3_ + HCOOH + 0.8H_2_O + Unknowns_Lignin/H^+^_	7.0 × 10^25^	25.7
	13	(C_5_H_8_O_4_)_*n*_ + H^+^ → Unknowns_Xylan_ + 1.5H_2_O + H^+^	5.5 × 10^16^	25.1
Corncob	14	Lignin + H^+^ → Unknowns_Lignin_ + H^+^	8.7 × 10^20^	20.0
	15	Lignin + H^+^ → Unknowns_Lignin/H^+^_	8.6 × 10^20^	20.0
	16	Corncob + H^+^ → (C_6_H_10_O_5_)_*n*_ + 1.12 (C_5_H_8_O_4_)_*n*_ + Lignin + H^+^	1.0 × 10^11^	0.0

a Calculated *E*_A_ values from Arrhenius fitting.

#### Diethyl ether

The condensation reaction of ethanol to diethyl ether is represented as an acid catalysed, reversible reaction in the mechanism. This sub-model also includes the protonation and deprotonation reactions of ethanol and water based on density functional theory.^40^ The model assumes that sulfuric acid fully dissociates in ethanol to give two hydrogen cations. It is essential that the competition for ethanol be described in the surrogate kinetic model.

#### Glucose

The conversion of glucose to ethyl levulinate is modelled to proceed through the intermediate ethyl glucoside. As discussed in section 3.1, this is the main experimentally observed pathway.

As shown later, the experiments reported in this study show mass balances of approximately 80%. As 100% of the reactant mass is not determined as products, the imposition of a conservation of mass in the modelling requires that species other than what is observed by measurement be considered. The formation of these species of unknown identity (humins and other soluble species of unknown identity) from lignocellulose ethanolysis is modelled by considering the formation of “unknowns”. From both glucose and ethyl glucoside, the model considers “unknowns_Glucose_” and “unknowns_Ethylglucoside_”, where the subscript indicates the source of the unknown species. The elemental composition of the humins has been determined experimentally to have a consistent approximate carbon to hydrogen molar ratio of 1 : 1.^13^ The formation of “unknowns” is accompanied by the formation of water in order to prescribe this C : H ratio as the elemental composition of “unknowns” in the model. The number of water molecules formed is derived by balancing the corresponding C : H ratio of the reactant molecules. The oxygen content of the “unknowns” is then inferred from oxygen content of the precursing molecule less the number of water molecules formed.

#### Cellulose

The cellulose molecule is modelled to depolymerise *via* two reaction pathways due to the presence of both ethanol and as the reaction proceeds, water: an acid ethanolysis pathway and an acid hydrolysis pathway. “Unknown” formation from cellulose ethanolysis is modelled through the formation of “unknowns_Cellulose_”.

#### Hemicellulose

Xylan, being the essential structural motif of hemicellulose in corncob,^55^ is used as a model compound for the hemicellulose portion of corncob. Thus, the hemicellulose sub-model is optimised to reproduce xylan experimental data. As there is little information on the reaction pathway of xylan to ethyl levulinate, xylan is modelled to form ethyl levulinate by the stoichiometrically balanced analogy to the reaction of cellulose to ethyl levulinate (eqn (4)). “Unknown” formation from xylan ethanolysis is modelled through the formation of “unknowns_Xylan_”.

#### Lignin

Lastly, the lignin sub-model represents the non-carbohydrate portion of the biomass and is optimised to the corncob experimental data. “Unknown” formation from lignin ethanolysis is modelled through the formation of “unknowns_Lignin_” and “unknowns_Lignin/H^+^_”, where “/H^+^” indicates that a hydrogen cation is consumed by the “unknowns” formation. The inclusion of both a catalytic and a non-catalytic pathway to “unknowns” allows their relative reactive flux to be optimised to represent the partial consumption of the sulfuric acid apparent from experiment (see Results & discussion).

#### Surrogate concept

The concept of a surrogate, a simple representation of the essential molecular composition of lignocelluloses, such as corncob, can be utilised to connect the compositional complexity and variability of real lignocelluloses to the compositionally discrete mono and polysaccharides. The surrogate concept allows for the additive learning of the chemical reaction kinetics and mechanism of real lignocelluloses.

In this way, corncob is modelled to decompose instantaneously into cellulose, hemicellulose and the non-carbohydrate portion of biomass in a mass ratio of 1.1 : 1.0 : 1.6 according to the biochemical composition determined for corncob by experiment (Table 2). The molecular compositions of cellulose and hemicellulose are defined as monomeric units of (C_6_H_10_O_5_)_*n*_ and (C_5_H_8_O_4_)_*n*_, consistent with the dehydrated repeating monomeric structures of glucopyranose and xylopyranose respectively. The molecular composition of corncob is defined according to the measured elemental ratio.^13^ For modelling purposes, one unit of corncob is defined so that it decomposes to form one monomer of cellulose. The relative number of xylan monomers formed is based on the mass ratio of cellulose to hemicellulose in corncob, assuming that all the hemicellulose consists of xylan. The composition of lignin is then inferred from the remaining elemental balance of the corncob.

**Table 2:** Biochemical analysis of corncob feedstock

Biochemical composition (% dry matter)					
Cellulose	Hemicellulose	Lignin	Ash	Extractives	Unknown
29.7	27.0	11.7	3.1	23.5	5.0

#### Modelling & optimisation

The kinetic modelling in this work uses Cantera,^56^ which is an open-source mathematical solver for problems involving chemical kinetics and thermodynamics. A homogeneous 0-D reactor model is used where all state variables are a function of time and thermodynamic equilibrium. The initial thermodynamic and chemical state is defined by declaration of pressure, temperature, and species mass fraction with the ideal gas equation of state employed.

The heating phase and isothermal phase of the reactor is modelled using a reactor network consisting of a reactor interconnected through a heat conducting wall with an external reservoir at the given reaction. At *t* = 0, the reactor is at 25 °C. The heat flux, *q*, through the wall is computed by *q* = *U*(*T*_reactor_ − *T*_reservoir_), where *U* is the heat transfer coefficient, *T*_reactor_ is the temperature of the reactor and *T*_reservoir_ is the temperature of reservoir. The heat transfer coefficient is optimised so that the temperature profile of the model reactor corresponds to the experimentally measured temperature profile of the reactors in the aluminium block.

A set of ordinary differential equations are numerically solved using Cantera^56^ to describe the concentrations of the species in the system as a function of time. An exemplar differential conversion equation for glucose is given by:7
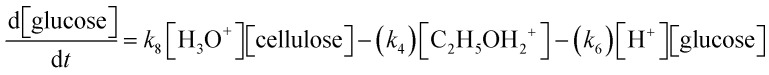
where *k*_8_ is the rate constant for the production of glucose from cellulose and *k*_4_ and *k*_6_ are the rate constants for the conversion of glucose to ethyl glucoside and “unknowns_Glucose_”.

All reactions progress according to their rate constant, *k*, with Arrhenius temperature dependence as, 
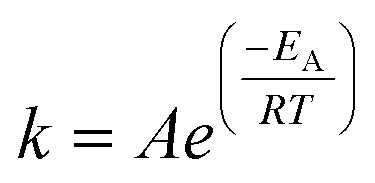
. Each rate parameter, *k*, is defined by an activation energy, *E*_A_, and a pre-exponent, *A*. Where applicable, the activation energy, *E*_A_, is given as the value derived according to section 2.6 (Fig. 6). All other activation energies and all pre-exponents are learned by a hierarchical, sub-model by sub-model optimisation procedure, where computed time resolved species fractions are compared to the time-resolved species fractions observed by the experimental data of this work, and the data of McNamara *et al.*^13^

The optimisation procedure is the phase 1 optimisation module of the MLOCK (Machine Learned Optimisation of Chemical Kinetics) algorithm as reported by Kelly *et al.*^57^ MLOCK generates a large library of kinetic model candidates (80 000) where the rate constant parameters, *A* and *E*_A_, of each reaction in the sub-model are randomly assigned across a *k* range up to the diffusion limit of 1 × 10^−13^ cm^3^ mol^−1^ s^−1^. MLOCK then computes the model at each condition for which experimental data is available and the error between the model candidate species fractions and those of experiment is calculated according to the least square error averaged across each experimental condition:8
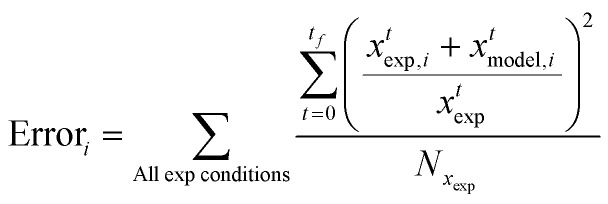
where *x*^*t*^_exp,*i*_ is the mass of species *i* measured experimentally after time *t*, *x*^*t*^_model,*i*_ is the mass of species *i* calculated by the model after time *t* and, *N*_*x*_exp__ is the number of experimental data points at that condition. The errors observed are used to set narrower ranges of *A* and *E*_A_ for each reaction through a bound refinement algorithm. A second library of 80 000 model candidates is generated at these refined ranges. A genetic seed scan is then performed centred on the resulting model candidate with the lowest error. The model candidate exhibiting the lower error function after these three iterations of optimisation is deemed to be the optimised model and the corresponding rate constants are selected as the empirical rate constants which allow for the best reproduction of the experimental data.

Each sub-model of Fig. 1 and Table 1 are optimised individually in hierarchical order to the corresponding experimental data based on the complexity of the feedstock molecule. Once derived, the rate constants for each sub-model are fixed in the overall system during subsequent optimisations in the described hierarchical order.

## 3. Results & discussion

For the purposes of identifying the main reaction pathways that should be supplied to the surrogate kinetic model, an experimental study is conducted to investigate the reaction mechanism responsible for the ethanolysis of glucose to ethyl levulinate.

This work then investigates the yield of the major reaction species (ethyl levulinate, diethyl ether and ethanol) produced from the model compounds, glucose, cellulose, xylan, and the biomass, corncob, across a range of temperatures.

A key trend investigated is the maximum yield of ethyl levulinate from the model compounds, as it defines the upper limit of ethyl levulinate production from biomass. There is a focus on overall production of ethyl levulinate from biomass, including its hemicellulose portion, rather than solely from cellulose.

Additionally, the yields of diethyl ether and ethanol are evaluated as functions of acid concentration, a factor that becomes increasingly important when transitioning from model compounds to biomass, where a partial catalytic behaviour is observed.

The trends observed in product yields from each model compound as a function of the reaction parameters are used to develop a surrogate kinetic model for corncob based on its specific biochemical composition.

### 3.1 Mechanistic study

**Fig. 2:**
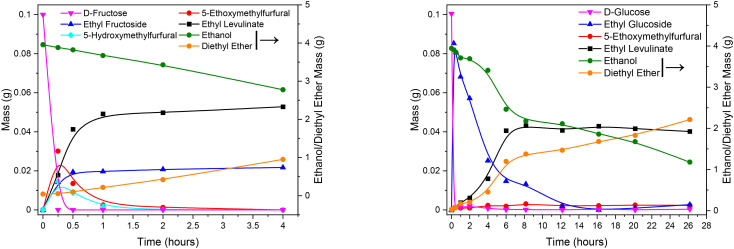
Mechanistic investigation of fructose (left) and glucose (right) in ethanol at 165 °C using 0.02 g H_2_SO_4_ and an initial hexose concentration of 0.1 g. Note the timescale of the glucose ethanolysis systems is an order of magnitude longer than that for the fructose ethanolysis system.

Time dependent species fraction evolutions for the acid catalysed reaction of fructose and glucose in ethanol at 165 °C are presented in Fig. 2. There are three major conclusions from the data of the mechanistic study:

(1) For fructose ethanolysis, at steady-state, average yields of 45.4 mass% ethyl levulinate are observed comparable to the glucose ethanolysis, where ethyl levulinate yields of 38.2 mass% are observed. In both cases, large quantities of ethanol are also observed to be consumed, with large quantities of diethyl ether formation observed in both systems.

(2) The time constant for steady state ethyl levulinate production from fructose is approximately a factor of ten faster than for ethyl levulinate production from glucose. This macroscopic observation indicates significant mechanistic differences between the two systems.

(3) Regarding intermediates and mechanism: both fructose and glucose are observed to be quickly depleted in concentration.

In the case of fructose, 5-hydroxymethylfurfural, 5-ethoxymethylfurfural and ethyl fructoside species are the major species observed, with only the ethyl fructoside species persisting as the system reaches steady sate at approximately 1 h.

In the case of glucose, it is observed that the glucose is immediately depleted from the system, with ethyl glucoside species being produced at fractions comparable to the initial glucose fraction. Only trace concentrations of 5-hydroxymethylfurfural and 5-ethoxymethylfurfural are observed. The ethyl glucoside species persist but are gradually diminished in concentration with time, coincident in time and quantity with an increase in concentration of ethyl levulinate, as the system proceeds to a steady state at approximately 12 h. This is in total contrast to the fructose system and provides compelling evidence that a different reaction process occurs for glucose and fructose ethanolysis with significant impact on the rate of product formation.

In the case of fructose, the observations and deductions are consistent with what is set out by Flannelly *et al.*^29^ in their mechanistic study. The fructose ethanolysis pathway appears to be fructose → 5-hydroxymethylfurfural → 5-ethoxymethylfurfural → ethyl levulinate, competing with an additional pathway of fructose → ethyl fructoside → 5-ethoxymethylfurfural → ethyl levulinate. Whereas for glucose the pathway appears to be glucose → ethyl glucoside → ethyl levulinate. Importantly, the presence of fructose is not observed in the ion chromatography of the experiments with glucose as starting material.

The pathway through which the glucose ethanolysis reaction proceeds is debated. The two most widely agreed upon routes are through the etherification into ethyl glucoside and the isomerization into fructose.^58–60^ Wang *et al.* calculated similar barriers for each pathway with the glucose to ethyl glucoside pathway having an energy barrier of 35.1 kcal mol^−1^, while the glucose to fructose pathway has an energy barrier is 39.8 kcal mol^−1^.^54^ However, ethyl glucoside is commonly observed as the main intermediate at the initial stage of glucose ethanolysis.^61,62^ It is not certain the route by which ethyl levulinate is then formed. It has been postulated that ethyl glucoside isomerises to form fructose or ethyl fructoside,^63–65^ although this is disputed in favour of the direct transformation to furan intermediates.^61^

On the basis of this evidence the basic reaction mechanism of major pathway appears to be glucose → ethyl glucoside → ethyl levulinate. As such, this sequence is supplied to the surrogate kinetic model.

### 3.2 Ethyl levulinate yields

Time dependent species fraction evolutions and steady state yields of ethyl levulinate production are determined for the sulfuric acid catalysed ethanolysis reactions of various feedstocks (glucose, cellulose, xylan, & corncob) at 150, 165, 180, 190, and 200 °C (Fig. 4). The steady state yields are compared to the maximum steady state yields identified for the same reaction conditions at 150 °C by McNamara *et al.*^13^

From Fig. 4, the rate of formation of ethyl levulinate from all feedstocks increases with increasing temperature. Significantly, the data shows that at a fixed initial reactant concentration, the steady state yield of ethyl levulinate formed from the ethanolysis of glucose, cellulose, xylan, and corncob is independent of reaction temperature. The maximum steady state yield of ethyl levulinate is dependent on the feedstock identity in decreasing order of glucose ≈ cellulose ≫ corncob ≫ xylan with average steady state yields (mass%) of 39.3, 39.1, 18.6, and 7.9%, respectively.

**Fig. 3:**
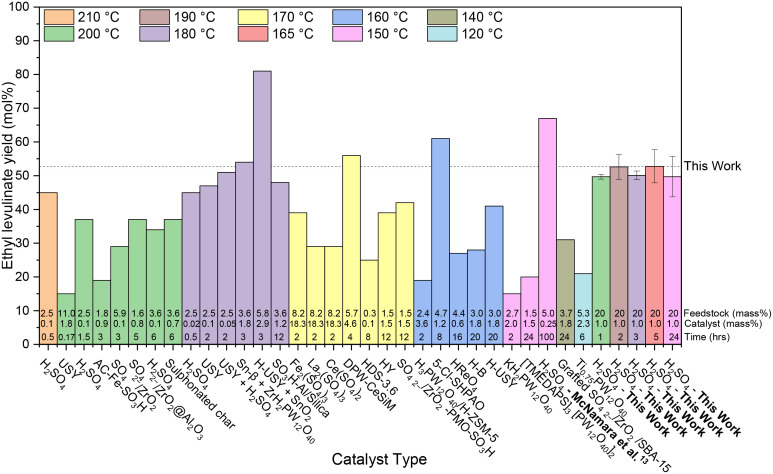
Literature review of experimental yields of ethyl levulinate from the ethanolysis of glucose using various catalyst types.^33,54,58,66–86^ All reaction systems use conventional heating and a one-pot process. The feedstock loading (mass%), catalyst loading (mass%), and reaction times are displayed at the bottom of each column. The dashed line represents the average steady state yield obtained in this work.

Fig. 3 analyses the “yield” of ethyl levulinate reported by literature studies as a function of reaction temperature, only considering glucose as the feedstock, and thus, all yields have been converted to molar yields. In the literature, there has been no coherent overarching attempt to systematically study the reaction conditions with a view to understanding their influence on the rate and/or yield of ethyl levulinate. Rather, the studies are more of a stand-alone nature, resulting in an unorganised ensemble of experimental parameters and associated observations.

In this work, Fig. 3 shows that the ethyl levulinate yields achieved from glucose are comparable or superior to those reported in the literature, despite using less severe reaction conditions. Notably, we obtained the highest yield recorded for a one-hour reaction time, under equivalent temperatures and catalyst loadings. In contrast to the work by McNamara *et al.*,^13^ carried out for the same reaction conditions, where a higher yield is obtained with a 5 mass% feedstock loading, our current work utilised a 20 mass% feedstock loading. This increase in feedstock loading is made to increase the concentration of ethyl levulinate in the reaction mixture, as distinct from the yield. The relationship between feedstock loading, yield, and ethyl levulinate concentration is further discussed in section 3.8.2, highlighting the efficiency of our method under these optimised conditions.

### 3.3 Ethyl levulinate production from biomass

The steady state yield of ethyl levulinate produced from the ethanolysis of corncob (18.6%) is approximately half that obtained from cellulose (39.1%). This is significantly higher than the pro-rata predicted yield of 11.6% based on the yield of ethyl levulinate from cellulose and the mass fraction of cellulose in corncob (29.7% dry matter). This information challenges the common literature assumption^87–90^ that all ethyl levulinate is derived from the cellulose portion of the biomass, indicating that ethyl levulinate may also be derived from the hemicellulose portion.

**Fig. 4:**
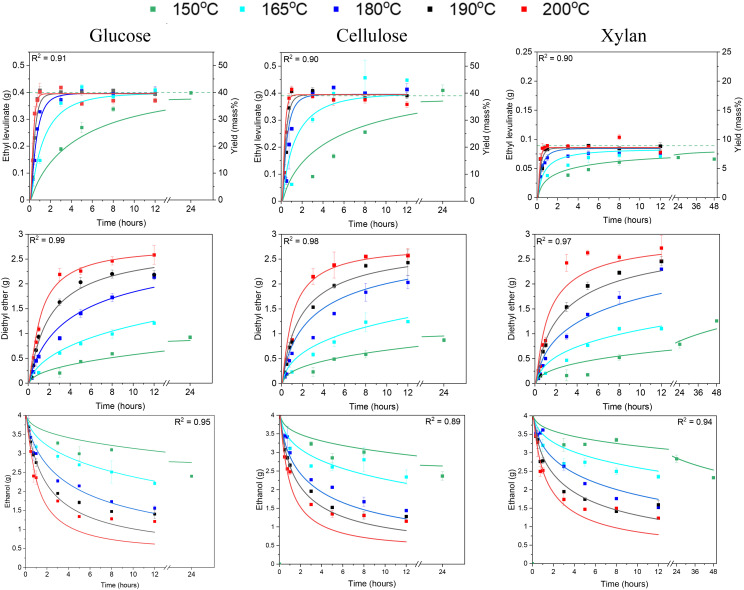
Ethyl levulinate and diethyl ether (g) produced from the sulfuric acid catalysed ethanolysis of glucose, cellulose, and xylan at 150, 165, 180, 190, and 200 °C. All reactions are carried out with 20 mass% feedstock and 1 mass% acid. The data points represent experimental data, and the lines represent the surrogate kinetic model. The dashed line is the maximum steady state yield identified for the same reaction conditions at 150 °C by McNamara *et al.*^13^

This hypothesis is investigated by specific experiments on ethanolysis of xylan, the results of which are shown in Fig. 4.

GC-MS measurements detected ethyl levulinate in the product mixture. The production of ethyl levulinate from xylan ethanolysis is of emphasised importance as this has not been previously reported. This insight significantly broadens the understanding of biomass potential for ethyl levulinate production, indicating that the economic value of biomass for biofuel production is not solely dependent on cellulose content.

Based on the steady state yields of ethyl levulinate formed from xylan and cellulose and their mass fractions in corncob, a steady state yield of ethyl levulinate of 14.6% is predicted from corncob. The observed yield is 4% higher than this predicted yield. This discrepancy may be due to an accumulation of experimental error, particularly given the high levels of uncertainty associated with biochemical analysis methods. Significantly increased yields of alkyl levulinate from xylan have been reported in the literature with the use of co-catalysts.^55^ Therefore, it may also be due to potential catalytic effects of the lignin, extractives and/or ash in the corncob increasing the yields of ethyl levulinate.

### 3.4 Catalyst consumption

**Fig. 5:**
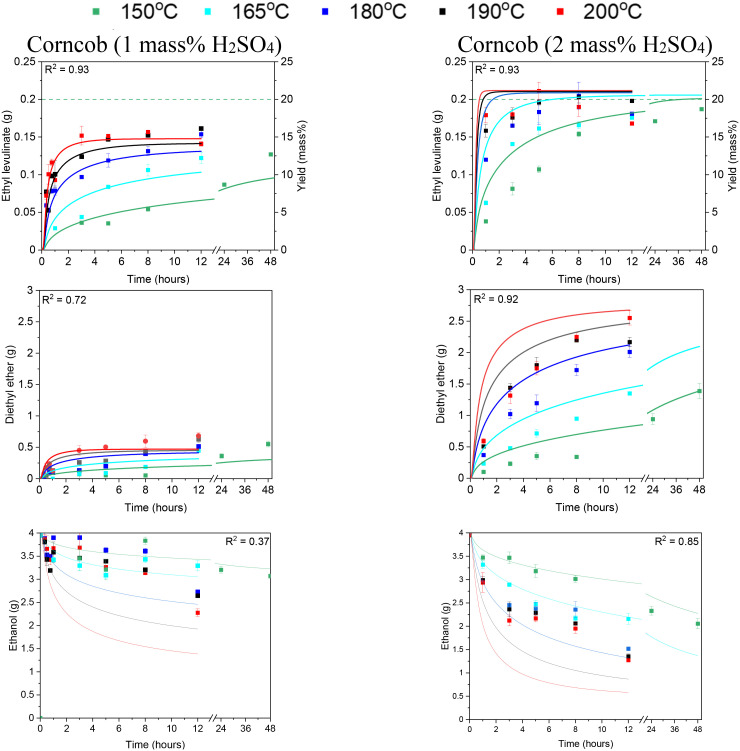
Ethyl levulinate (g) and diethyl ether (g) produced from the sulfuric acid catalysed ethanolysis of 20 mass% corncob for 1 and 2 mass% acid at 150, 165, 180, 190, and 200 °C. The data points represent experimental data, and the lines represent the surrogate kinetic model. The dashed line is the steady state yield identified for the same reaction conditions at 150 °C by McNamara *et al.*^13^

A common assumption in the alcoholysis literature is that of perfect catalysis, where the catalytic agent is regenerated stoichiometrically. If the catalyst is not consumed in the reaction the steady state yield would not be affected by the initial mass of sulfuric acid. The data in Fig. 5 shows that the steady state yield of ethyl levulinate formed from the ethanolysis of corncob is dependent on the feedstock to sulfuric acid ratio. Reaction conditions of 1 mass% acid and 20 mass% corncob (20 : 1 feedstock to acid ratio), achieved an average steady state ethyl levulinate yield of 14.7%, a reduced yield compared to the maximum steady state yield of 19.6 (±4.8)% identified by McNamara *et al.* under identical conditions.^13^ This is an important finding as it implies the catalyst is partially consumed in competitive processes alongside the ethanolysis reaction.

The steady state yield of ethyl levulinate from corncob is limited by the feedstock to sulfuric acid ratio due to the partial catalytic nature of corncob ethanolysis. Below a certain threshold quantity of sulfuric acid, the reaction will not proceed to completion, limiting the yield. This decrease in catalytic activity in the corncob reaction system is also reflected in the rate of formation of diethyl ether. As the formation of diethyl ether is both a function of initial sulfuric acid and ethanol concentrations, the decrease in diethyl ether production indicates that for the real lignocellulosic biomass, corncob, the sulfuric acid is being consumed in some irreversible process. A decreased feedstock to sulfuric acid ratio is required to achieve the maximum steady state yield of ethyl levulinate formed from corncob at these reaction conditions.

Thus, additional experiments are performed to identify an initial sulfuric acid concentration sufficient in achieving the maximum steady state yield of ethyl levulinate. Fig. 5 shows that a sulfuric acid concentration of 2 mass% (10 : 1 feedstock to acid ratio) facilitates the formation of an average steady state yield of 18.6%. This is consistent with the maximum steady state yield of ethyl levulinate from corncob identified by McNamara *et al.*^13^

### 3.5 The “ether effect”

Diethyl ether formation from ethanolysis of lignocellulose has been noted by a few prior studies^30,35,37^ but not studied quantitatively in conjunction with ethyl levulinate formation. Diethyl ether is an unavoidable by-product of acid catalysed ethanolysis reactions (eqn (5)) and is formed in the parallel acid-catalysed self-condensation reaction of the solvent, ethanol. Thus, the rate of formation of diethyl ether from all reaction systems increases with both increasing temperature and increasing sulfuric acid concentration. The steady state yield of diethyl ether is not reached in the time scale investigated. For the model compounds, no significant difference in diethyl ether production is observed indicating that the level of catalyst consumption is the same across glucose, cellulose, and xylan. In Fig. 5, corncob with 1 mass% acid shows a significant drop in diethyl ether produced, as discussed in section 3.4. At the increased sulfuric acid concentration of 2 mass% acid, the mass of diethyl ether is greater at each timepoint compared to corncob at 1 mass% acid but remains less than the mass of diethyl ether produced from the model compounds at 1 mass% acid. While 2 mass% acid is sufficient to form the maximum steady state yield of ethyl levulinate from corncob, the concentration of sulfuric acid in the system is less than in the model compound reactions indicating that more than half the initial sulfuric acid is consumed irreversibly by the corncob.

As the solvent, ethanol, is present in excess, ethanol, diethyl ether, and water are the largest components in the product mixture, showing the importance of the “ether effect” which is crucially neglected in literature studies. Diethyl ether is an undesired co-product in the acid-catalysed ethanolysis of biomass. It is therefore sought to minimise the concentration of sulfuric acid and reaction temperature to likewise minimise diethyl ether production. Once the yield of ethyl levulinate reaches its maximum value, any further reaction only increases diethyl ether concentration and consumes ethanol. As diethyl ether and water may need to be partially or fully removed, particularly if the focus is to be a usable fuel mixture, the amount of diethyl ether produced should be minimised. A sub-model of this parallel process has therefore been integrated into the surrogate kinetic model to allow for the determination of the optimal reaction temperature, initial concentration of sulfuric acid, and reaction time to produce a maximum yield of ethyl levulinate and minimum yield of diethyl ether in the product mixture.

When corncob is used as the feedstock, the conversion of ethanol to diethyl ether appears to coalesce at higher temperatures. As the temperature increases, the ethanol consumption is expected to increase due to a higher reaction rate. However, literature indicates that higher temperatures also lead to increased humin formation,^91^ which results in higher acid consumption. An increased acid consumption reduces the overall rate of reaction. Consequently, although higher temperatures accelerate the reaction rate, the concurrent decrease in acid concentration mitigates this effect. This phenomenon may explain the similar ethanol consumption rates at higher temperatures.

### 3.6 Activation energies

**Fig. 6:**
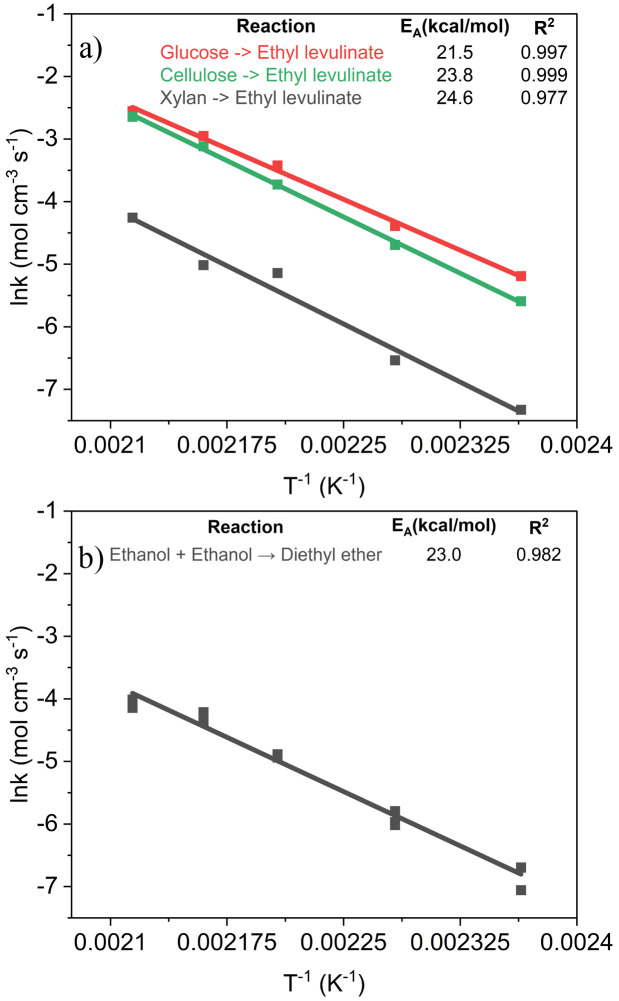
Arrhenius rate constants *vs. T*^−1^ with apparent activation energies for the global reactions to a) ethyl levulinate and b) diethyl ether.

The apparent activation energy for the reaction of ethanol to diethyl ether based on the diethyl ether formed during the ethanolysis of glucose, cellulose, and xylan is 23.0 kcal mol^−1^. The apparent activation energies for the formation of ethyl levulinate from glucose, cellulose and xylan are 21.5, 23.8, and 24.6 kcal mol^−1^, respectively (Fig. 6). A quantum chemical investigation by Wang *et al.* into the reaction pathways and mechanism of glucose conversion to ethyl levulinate catalysed by a Brønsted acid in solution phase found the primary thermodynamic and kinetic pathway had an energy barrier of 20.8 kcal mol^−1^.^54^ Literature values for the apparent activation energy of glucose to ethyl levulinate range from 15.5–29.5 kcal mol^−1^.^30–36,92,93^ The apparent activation energies are input into the surrogate kinetic model where applicable to accurately model the temperature dependence of the system.

### 3.7 Surrogate kinetic model

Fig. 1 shows the surrogate reaction mechanism for the formation of ethyl levulinate, diethyl ether, and ethanol developed from experimental measurements reported in this study and in the literature. Experimentally determined product mass fractions are coupled with the surrogate kinetic model to obtain the empirical kinetic constants (Table 1) as described in section 2.7. The temporal output of the model for each experimentally investigated reaction condition is shown alongside the experimental results in Fig. 2 and 3.

The accuracy of the surrogate kinetic model in calculating the mass of ethyl levulinate, diethyl ether, and ethanol produced is measured by the coefficient of determination (*R*^2^) for each sub-model at all reaction conditions. The *R*^2^ values of the model for ethyl levulinate, diethyl ether, and ethanol production from glucose, cellulose and corncob are given in Fig. 2 and 3 with an overall *R*^2^ of 0.88 averaged across each sub-model, establishing that the surrogate kinetic model achieves a good fit.

Ethanol notably exhibits the poorest fit compared to the other products. It is hypothesised that this is due to some of the unknown species possibly consuming ethanol in their formation, which is not accounted for in the reaction scheme.

### 3.8 Ethanolysis process predictions

**Fig. 7:**
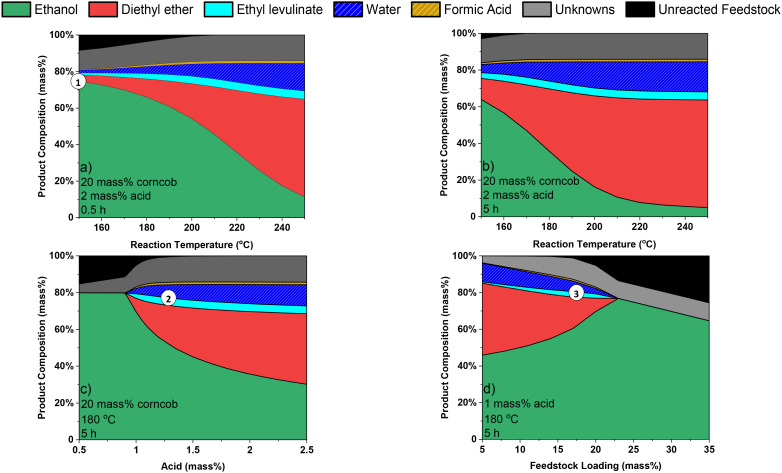
Surrogate kinetic model predicted product mixtures from corncob ethanolysis as a function of a) temperature at 0.5 h, b) temperature at 5 h, c) acid concentration, and d) feedstock loading at fixed corncob : acid mass ratio. The inscribed labels show the 1) temperature which minimises diethyl ether formation, 2) acid concentration which maximises the yield of ethyl levulinate and 3) feedstock loading which maximises the concentration of ethyl levulinate at the given reaction conditions.

Analysis of experimental results are inherently limited to the specific conditions investigated. The surrogate kinetic model facilitates the extrapolation of the experimental results to predict the product mixture composition formed at varying reaction times, temperatures, and reactant concentrations. To demonstrate this, an analysis of the surrogate kinetic model, at exemplar conditions as continuous functions of temperature, sulfuric acid concentration, and feedstock loading is presented in Fig. 7.

#### 3.8.1 Temperature

Temperature and reaction time are intrinsically linked. Higher temperatures reach the same steady state yield of ethyl levulinate earlier than lower temperatures, which simply require longer reaction times but reach the same state yield. The energy costs in attaining these trade-off reaction conditions are important to assess the economic performance of the process.

The temperature dependence of the composition of the product mixture is modelled at 0.5 hours (Fig. 7a) and 5 hours (6b). The reaction condition which minimises diethyl ether formation significantly reduces the yield of ethyl levulinate (1). Overall, no considerable minimisation effect on ethanol conversion relative to ethyl levulinate concentration can be achieved by varying the temperature–time parameter. The magnitude by which the rate of reaction of ethyl levulinate formation and diethyl ether formation are affected by temperature is similar, as evidenced by their derived activation energies. Thus, the relative yields of the products are not significantly influenced by the reaction temperature.

At the temperatures where diethyl ether formation after 0.5 hours is equal to the diethyl ether formation after 5 hours, the yield of ethyl levulinate is approximately equal across all temperatures. The temperatures at which these conditions occur are consistently ∼35 °C higher at 5 hours than at 0.5 hours.

#### 3.8.2 Feedstock loading & acid concentration

Feedstock loading and sulfuric acid concentration are also intrinsically linked. Higher feedstock loadings achieve a higher concentration of ethyl levulinate while higher sulfuric acid concentrations are consequentially required to maintain the catalysis of the reaction for real-world lignocellulosic biomass. Analysis of the cost balance between these factors is also important to assess the process.

After 5 hours at 20 mass% feedstock and 180 °C, the model predicts the maximum yield of ethyl levulinate to be achieved at 1.35 mass% acid (2). At the same conditions for higher mass% acid, an equivalent yield of ethyl levulinate is achieved while producing more diethyl ether. Thus, improved knowledge of the extent of acid consumption in real biomass feedstocks is essential to the development of the ethanolysis process but has been rarely assessed in the literature.

The model can inform in this regard, after 5 hours at 1 mass% acid and 180 °C, the model predicts a maximum concentration of ethyl levulinate to be achieved at 17.5 mass% feedstock (3). Fig. 7d shows as feedstock loading increases, concentration of ethyl levulinate increases up to a maximum before decreasing to a negligible concentration as more feedstock is added, as the catalytic property of the system has ceases entirely at a critical feedstock to acid ratio. This phenomenon is due to the consumption of acid effect identified in this study.

Overall, the surrogate kinetic model allows for species fraction predictions to be studied as a function of reaction variables. In contrast to popular data-based modelling methods such as the popular design of experiment, this alternative approach is grounded in physics and is authentic to reaction mechanistic chemistry using steady state thermochemical constants derived from experiment and mass conserved reaction equations to allow for physics-based prediction of reaction trends.

## Conclusions

This work establishes the temperature dependence of the ethanolysis of glucose, cellulose, xylan, and corncob, catalysed by sulfuric acid. It is shown that prolonged reaction time and increased temperature promote the conversion to ethyl levulinate. The steady yield of ethyl levulinate produced from glucose, cellulose, xylan, and corncob is independent of temperature. Maximum steady state yields (mass%) of ethyl levulinate of 39.3, 39.1, 7.9, and 18.6% are produced from glucose, cellulose, xylan, and corncob, respectively. These yields are comparable or superior to those in the literature for equivalent or less severe conditions. The finding of a significant ethyl levulinate yield from xylan is novel and provides new understanding of the mechanism of the process in biomasses. This finding implies that the hemicellulose portion of any biomass would contribute to the total yield of ethyl levulinate, which contradicts common understanding that yield is proportional to only the cellulose fraction of the biomass.

In contrast to the situation of the model compounds, the steady state yield of ethyl levulinate formed from corncob is shown to be dependent on the initial concentration of sulfuric acid. A corncob : acid ratio of 20 : 1 resulted in a reduced yield of ethyl levulinate, consistent with a thesis that sulfuric acid in some consumed in some irreversible process. A decreased corncob : acid ratio of 10 : 1 is identified as a sufficient initial mass of sulfuric acid to facilitate the formation of the maximum yield of ethyl levulinate.

At all conditions, for all feedstocks, the formation of diethyl ether is observed to be a major process, where the steady state yield of diethyl ether is not reached at the conditions investigated. The apparent activation energy of the global reaction to diethyl ether is 23.0 kcal mol^−1^, similar to that of the global reaction to ethyl levulinate from glucose, cellulose, and xylan at 21.5, 23.8, and 24.6 kcal mol^−1^ respectively.

The study introduces the concept of an elemental mass conserved, hierarchical, surrogate chemical kinetic model to describe the ethanolysis of corncob based on its biochemical composition of cellulose, hemicellulose, and lignin. To inform the model, a mechanistic study is performed analysing the comparative reaction pathways apparent in the ethanolysis of fructose and glucose. This work establishes higher ethyl levulinate yields are possible using fructose (45.4 mass%) as a reactant compared to using glucose (38.2 mass%) as reactant with the rate of formation of ethyl levulinate an order of magnitude slower from glucose than from fructose. Species fractions measurements show that glucose is rapidly converted to ethyl glucoside, which is subsequently converted to ethyl levulinate. Fructose was not observed as an intermediate in the ethanolysis of glucose. For fructose, species fractions measurements show 5-hydroxymethylfurfural, 5-ethoxymethylfurfural and ethyl fructoside as competitive major intermediate species.

This information is used to describe the reaction pathways in the surrogate kinetic model which has an *R*^2^ value of 0.88 for the reproduction of ethyl levulinate, diethyl ether and ethanol fractions across all conditions and feedstocks. The model has developed a baseline understanding of the system and for the first time concurrently models the production of ethyl levulinate along with the other major products of the system from each of the main sugar portions of the biomass. In contrast to popular data-based modelling methods, this alternative approach grounded in physics and reaction mechanistic chemistry uses thermochemical constants derived from experiment and mass conserved reaction equations to allow for the predictivity of reaction trends. It is recommended as an alternative promising method to aid in understanding complex mechanistic processes and in the design of chemical engineering processes.

## Author contributions

The manuscript was written through contributions of all authors. All authors have given approval to the final version of the manuscript.

## Conflicts of interest

There are no conflicts to declare.

## Supplementary Material

RE-010-D4RE00378K-s001

## Data Availability

The data supporting this article have been included as part of the ESI.†
