# Supplementary material for: A hierarchical surrogate approach to biomass ethanolysis reaction kinetic modelling
Source: React Chem Eng. 2024 Nov 13;10(2):344–59. doi: 10.1039/d4re00378k (PMC12178304; doi:10.1039/d4re00378k)
Supplement: RE-010-D4RE00378K-s001 [file RE-010-D4RE00378K-s001.pdf]

Supplementary Information (SI) for Reaction Chemistry & Engineering.
This journal is © The Royal Society of Chemistry 2024

Supplemental Information:

A Hierarchical Surrogate Approach to Biomass Ethanolysis Reaction Kinetic Modelling

S1. Biochemical Composition of Corncob Data

S1.1 Aldoses

	Glucan	Xylan	Arabinan	Galactan	Mannan	Rhamnan
Wet-Chemical Data (% Dry Matter)						
Average	**29.68**	**24.19**	**2.11**	**0.53**	**0.08**	**0.04**
Repeat 1	29.81	24.27	2.12	0.53	0.08	0.04
Repeat 2	29.56	24.11	2.10	0.53	0.08	0.04
Std. Dev.	0.17	0.11	0.01	0.00	0.00	0.00

S1.2 Total Sugars and Sugar Classes

	Hexosans	Pentosans	Total Sugars
Wet-Chemical Data (% Dry Matter)			
**Average**	**30.33**	**26.30**	**56.63**
Rep 1	30.45	26.39	56.84
Rep 2	30.21	26.21	56.42
Std. Dev.	0.17	0.12	0.29

S1.3 Lignin Content

	Klason Lignin	Acid Soluble Lignin	Acid Insoluble Residue
Wet-Chemical Data (% Dry Matter)			
**Average**	**8.63**	**3.09**	**9.00**
Rep 1	8.62	3.23	8.91
Rep 2	8.64	2.96	9.09
Std. Dev.	0.02	0.19	0.12

S1.4 Ash and Extractives

	Ash	Acid Insoluble Ash	Full Extr.	Water Soluble Extr.	Ethanol Soluble Extr.	Water Inoluble, Ethanol Soluble Extr.
Wet-Chemical Data (% Dry Matter)						
**Average**	**3.08**	**0.37**	**23.44**	**21.55**	**7.63**	**1.89**
Rep 1	3.34	0.30	23.72	21.85	7.54	1.87
Rep 2	2.82	0.44	23.16	21.26	7.72	1.91
Std. Dev.	0.37	0.10	0.40	0.42	0.13	0.03

S1.5 Sugars in Water Extract

	Glucose	Fructose	Mannose	Galactose	Rhamnose
**Free Sugars (% Dry Matter)**					
**Average**	**0.075**	**0.032**	-	**0.028**	-

Rep 1	0.076	0.027	-	0.030	-
Rep 2	0.074	0.037	-	0.025	-
Std. Dev.	0.002	0.007	-	0.004	-

S1.6 Total Lignocellulosic Composition

	Cellulose	Hemicellulose	Lignin	Total Lignocellulose
Wet-Chemical Data (% Dry Matter)				
**Average**	**29.68**	**26.95**	**11.72**	**68.35**
Rep 1	29.81	27.16	11.71	68.68
Rep 2	29.56	26.74	11.73	68.03
Std. Dev.	0.17	0.30	0.01	0.46
Adjusted Cellulosic Content for D3 RINS (% Dry Mass Ash-Free Basis)				
**Average**	**30.62**	**27.81**	**12.09**	**70.52**
Rep 1	30.76	28.02	12.08	70.86
Rep 2	30.50	27.59	12.10	70.19
Std. Dev.	0.18	0.31	0.01	0.47

S2. Calibration Curves for Gas Chromatography

(a)

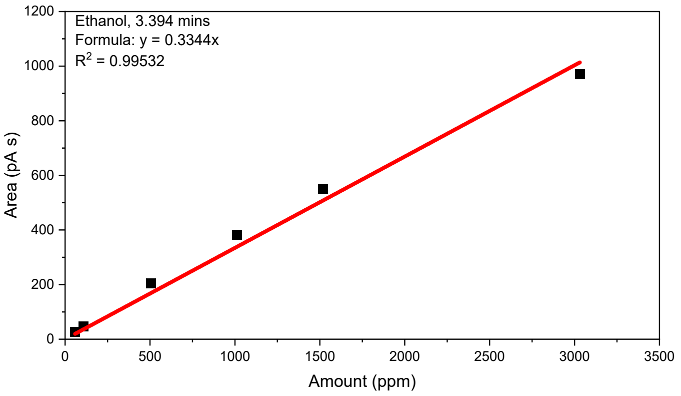

(b)

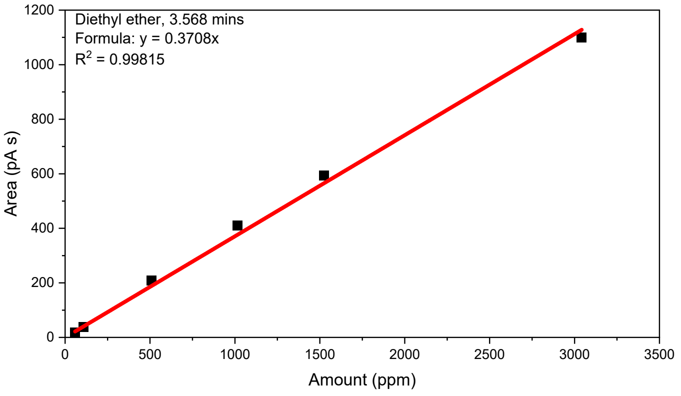

(c)

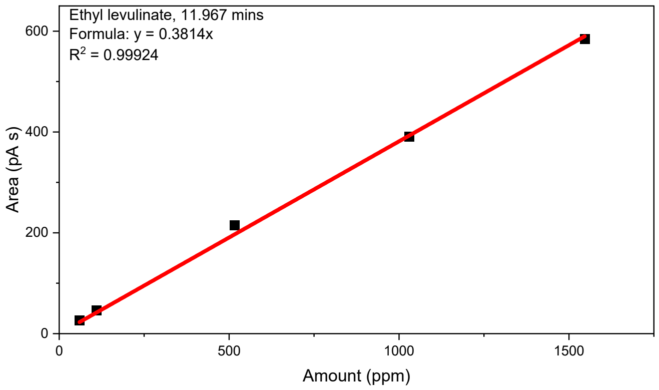

Figure S1. Gas chromatography calibration curve for the major species in the system (a) ethanol, (b) diethyl ether, and (c) ethyl levulinate
